# Supplementary material for: Finding gaps in the national electric vehicle charging station coverage of the United States
Source: Nat Commun. 2025 Jan 27;16:561. doi: 10.1038/s41467-024-55696-8 (PMC11772696; doi:10.1038/s41467-024-55696-8)
Supplement: Supplementary file 1 — Supplementary Information [file 41467_2024_55696_MOESM1_ESM.pdf]

Supplementary Information
Finding Gaps in the National Electric Vehicle Charging Station Coverage of the United States

Lily Hanig[1, 2, *], Catherine Ledna[2], Destenie Nock[1, 3], Corey D. Harper[3, 4], Arthur Yip[2], Eric Wood[2], and C. Anna Spurlock[5]

[1]Engineering and Public Policy, Carnegie Mellon University, 5000 Forbes Ave., Pittsburgh PA, 15213
[2]National Renewable Energy Laboratory, 15013 Denver West Parkway, Golden CO, 80401
[3]Civil and Environmental Engineering, Carnegie Mellon University, 5000 Forbes Ave., Pittsburgh PA, 15213
[4]Heinz school of Policy and Information Systems, Carnegie Mellon University, 5000 Forbes Ave., Pittsburgh PA, 15213
[5]Lawrence Berkeley National Laboratory, 1 Cyclotron Rd., Berkeley CA, 94720
[*]Corresponding Author, lily.r.hanig@gmail.com

Table of Contents

Supplementary Figure 1. **County-level consecutive coverage with & without proprietary chargers.** 1

Supplementary Figure 2. **County-level consecutive coverage distance sensitivity.** 2

Supplementary Figure 3. **Medium and heavy duty charging stations.** 3

Supplementary Figure 6. **Medium-duty consecutive coverage.** 4

Supplementary Figure 5. **Heavy-duty consecutive coverage.** 5

Supplementary Figure 6. **Long-distance charging station access equity analysis.** 6

Supplementary Figure 7. **Data processing flowchart.** 7

Supplementary Figure 8. **Flowchart of Methods.** 8

Supplementary Discussion 9

Supplementary References 10

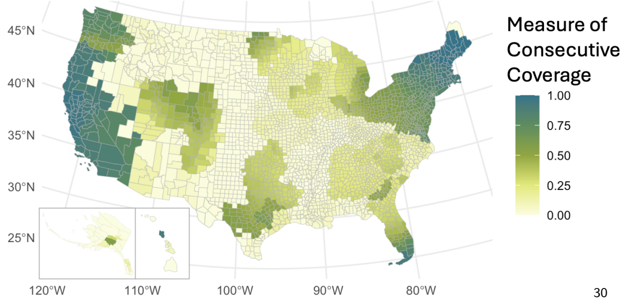

(a) Minimum Viable Coverage (no Tesla stations)

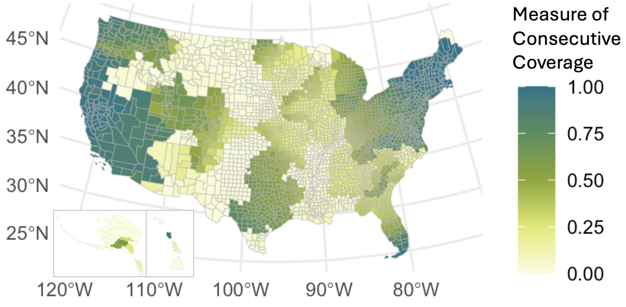

(b) Minimum Viable Coverage (with Tesla stations)

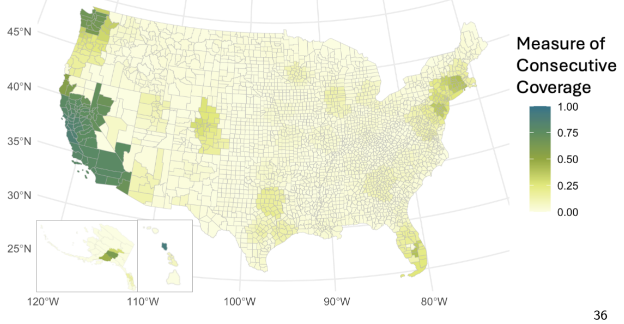

(c) Fast Charger Coverage (no Tesla stations)

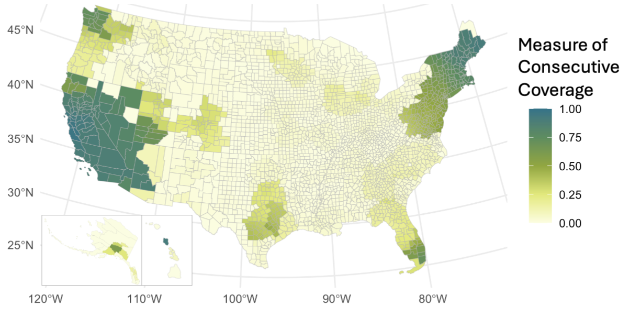

(d) Fast Charger Coverage (with Tesla stations)

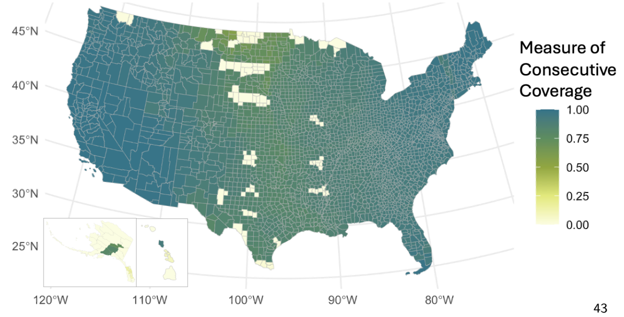

(e) Alternative Fuel Corridors Reach NEVI-Compliant Status (no Tesla stations)

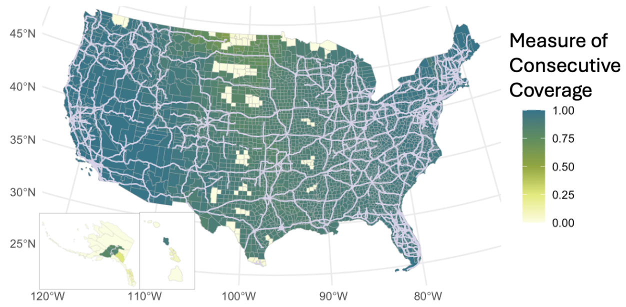

(f) Alternative Fuel Corridors Reach NEVI-Compliant Status (with Tesla stations)

Supplementary Figure 1. **County-level consecutive coverage with and without proprietary chargers.** Map of consecutive coverage at the county level. a, c, and e show maps for the three scenarios excluding Tesla charging stations. b, d, and f shows maps for the three scenarios including Tesla charging stations. Tesla is capable of installing Magic Docks that make their Supercharger charging stations accessible to non-Tesla Electric Vehicles, as they have begun to with Ford, General Motors, and Rivian[1]. If Tesla were to make their chargers universally accessible, the counties surrounding cities in the Great Plains (Texas through Montana) benefit the most, with some areas increasing from very low coverage to 50% coverage with access to Tesla chargers. The second row of maps shows the Fast Charger Coverage (i.e., four or more DC fast chargers at a station in 2023). The third row shows the coverage if Alternative Fuel Corridors reach National Electric Vehicle Infrastructure (NEVI) program compliance. The Northeast benefits substantially (146 additional road segments have charging station access) if Tesla charging stations are made universally accessible, with coverage increasing from 0%–30% without Tesla chargers to 50%–100% throughout the Northeast for fast charger coverage. Southern Florida, the Houston area, eastern California, and Nevada also increase coverage.

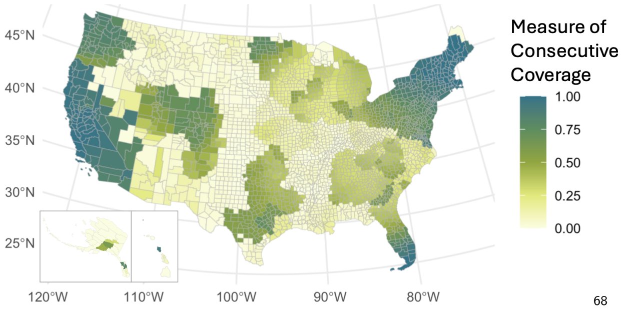

(a) Minimum Viable Coverage (within 400 miles)

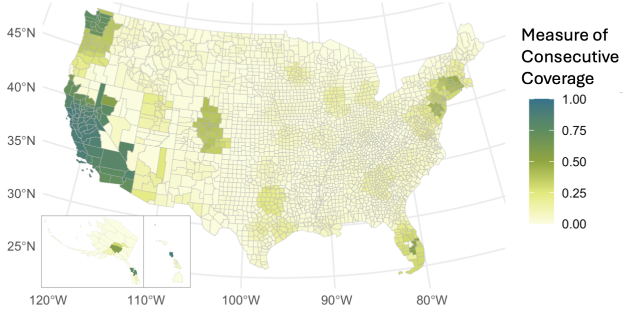

(b) Fast Charger Coverage (within 400 miles)

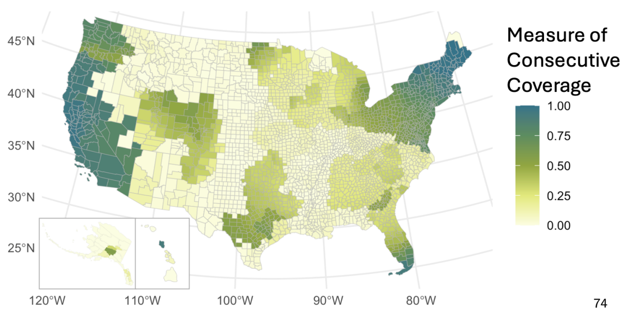

(c) Minimum Viable Coverage (within 500 miles)

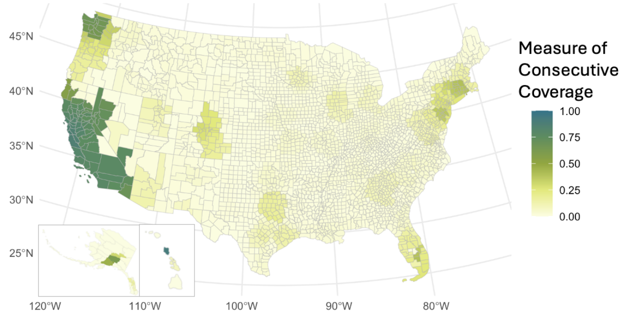

(d) Fast Charger Coverage (within 500 miles)

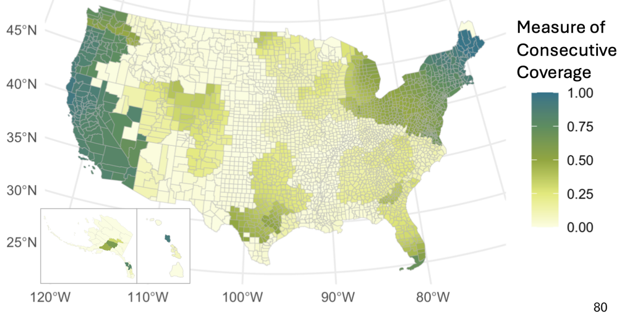

(e) Minimum Viable Coverage (within 600 miles)

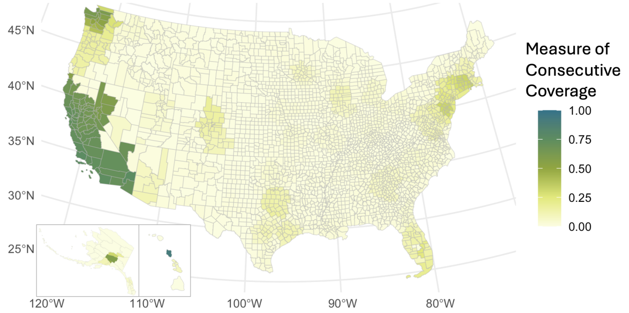

(f) Fast Charger Coverage (within 600 miles)

Supplementary Figure 2. **County-level consecutive coverage distance sensitivity.** Map of consecutive coverage at the county level. The top row (a and b) shows maps for coverage within 400 miles of each county population center, the middle row (c and d) shows maps for coverage within 500 miles (the baseline case), and the bottom row (e and f) shows coverage within 600 miles. For minimum viable coverage, increasing the range considered shows a similar coverage map but lowers the magnitude, particularly for urban counties in the central United States (e.g., Denver, Dallas, Chicago) due to the expanded range including more rural regions with few charging stations. Michigan gained in minimum viable coverage when considering 600 miles due to the expanded range encompassing charging stations on the East Coast. The general pattern of coverage does not shift for minimum viable coverage or fast charger coverage with the West Coast, South Florida, and the Northeast still reaching high coverage, and much of the middle of the country left with low coverage across the distance sensitivity cases.

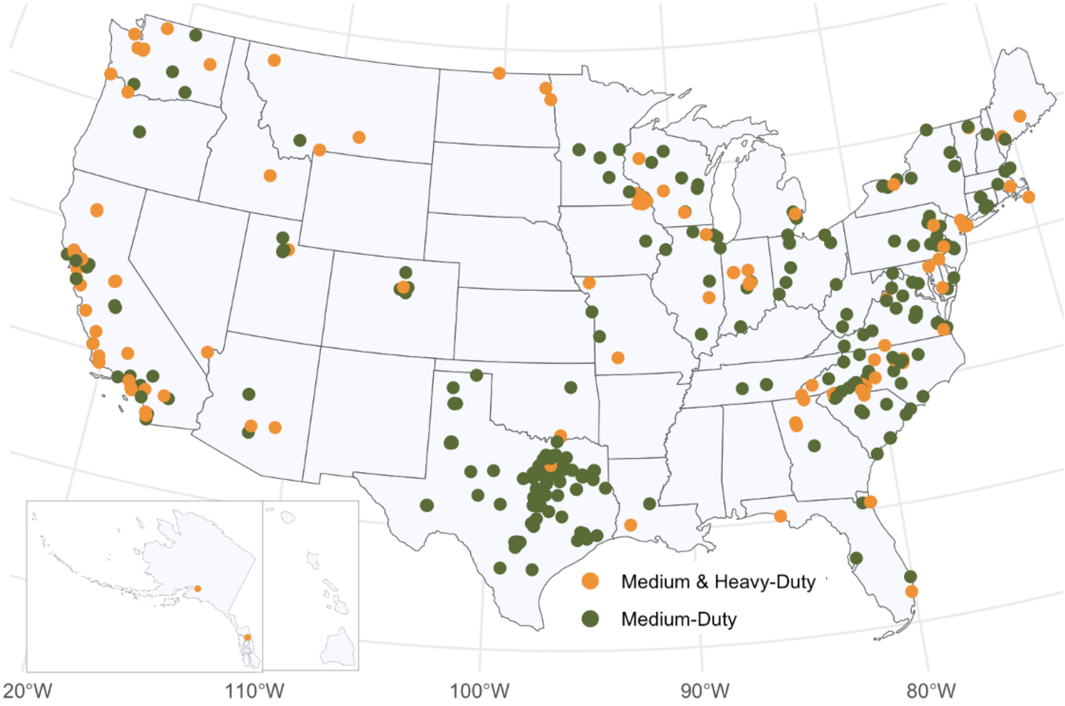

Supplementary Figure 3. **Medium and heavy duty charging stations.** Public electric vehicle chargers for medium- and heavy-duty trucks in the United States as of April 9, 2024. The orange dots show all stations that have a maximum vehicle class of heavy-duty (allowing for medium and heavy-duty). The dark green dots show all stations with a maximum medium-duty vehicle class. Medium- and heavy-duty trucks have different vehicle sizes and trailer requirements than light-duty vehicles, requiring larger station designs[2] and in some cases higher-powered charging[3]. There are 400 medium-duty (Class 3–5, or a gross vehicle weight rating [GVWR] of 10,001–19,500 pounds) and 100 heavy-duty (Class 6–8, or a GVWR of 19,501 pounds and above) public Electric Vehicle charging stations in the United States as of April 2024, shown in Fig. 3[4], compared to the 62,000 public charging stations for light-duty vehicles. We exclude private depot chargers.

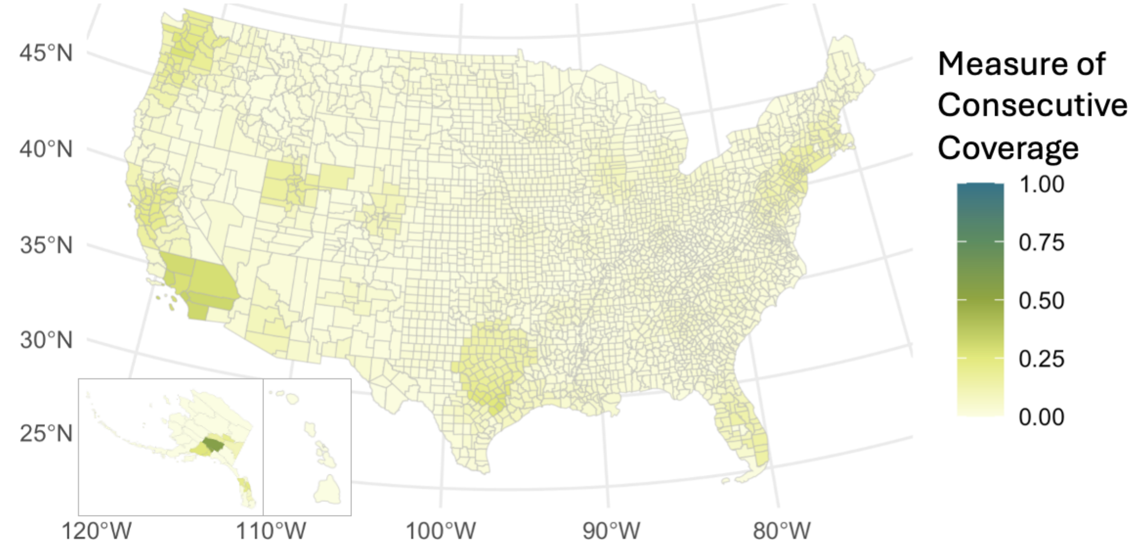

Supplementary Figure 4. **Medium-duty consecutive coverage.** Charging station consecutive coverage of medium-duty trucks for all Level 2 and direct current fast chargers within 1 mile of an National Highway System road, Minimum Viable Coverage scenario. California reaches the highest state-level charging station coverage for both medium- and heavy-duty coverage (24% in both cases), while 37 states have state-level coverage below 10% for medium duty and 40 states have coverage below 10% for heavy duty. Charging station access and investment for freight lags significantly behind that of light-duty vehicles. Public charging station access for trucks lags behind charging station access for light-duty vehicles and will probably lag even further following the build-out of the National Electric Vehicle Infrastructure program.

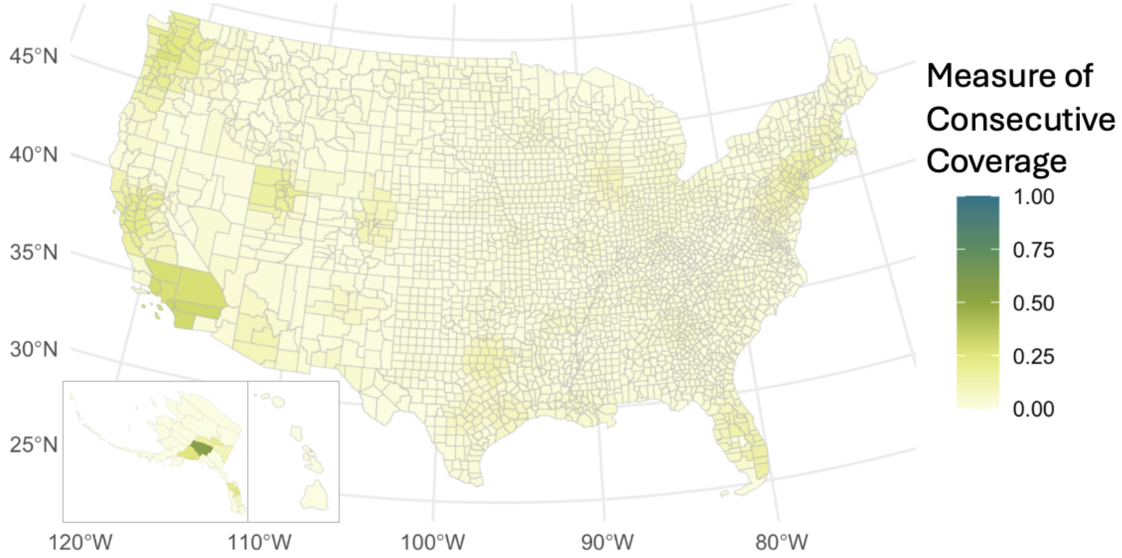

Supplementary Figure 5. **Heavy-duty consecutive coverage.** Charging station consecutive coverage of medium- and heavy-duty trucks for all Level 2 and direct current fast chargers within 1 mile of an National Highway System road, Minimum Viable Coverage scenario.

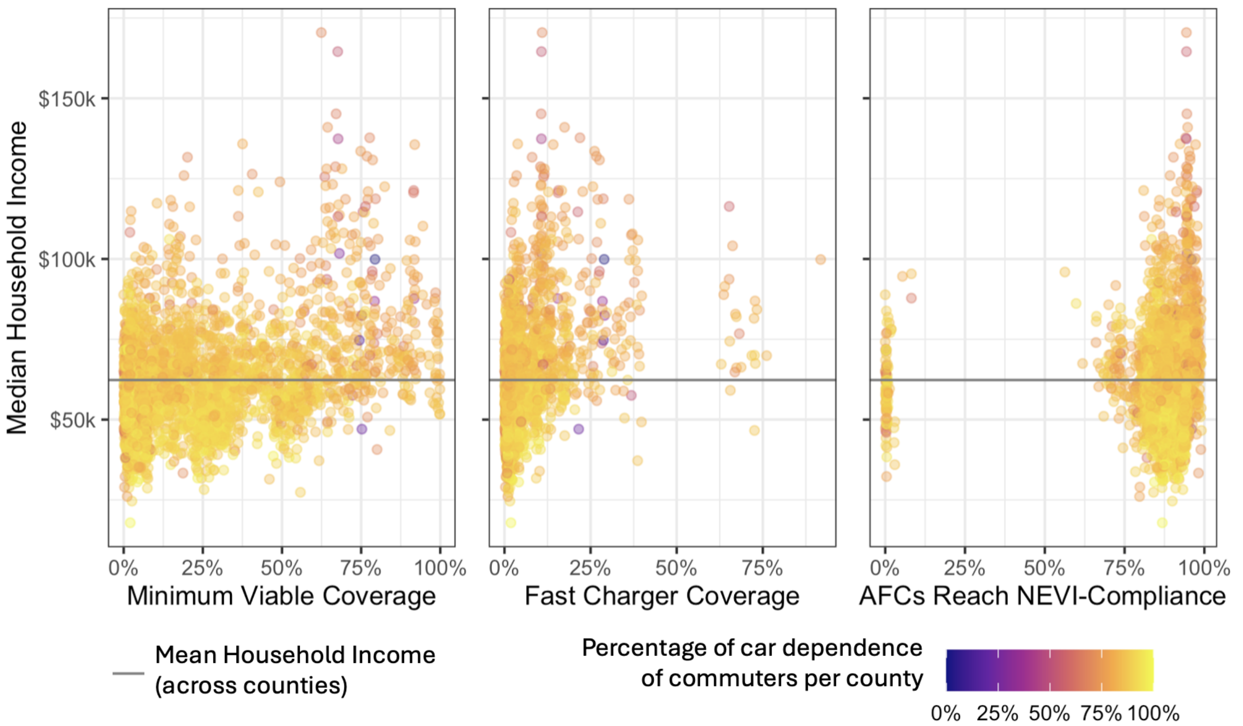

Supplementary Figure 6. **Long-distance charging station access equity analysis.** Median household income of each county in the United States versus charging station coverage for minimum viable coverage, fast charger coverage, and when Alternative Fuel Corridors (AFCs) reach National Electric Vehicle Infrastructure (NEVI) program compliant status. The color represents the percentage of residents in each county that commute by car, van, or light-duty truck. The current state of minimum viable coverage in the United States trends slightly higher with median household income. Very urban counties with commuter car dependence below 25% have long-distance minimum viable charging station coverage at or above 50%; minimum viable coverage is higher among urban counties despite lower car dependence overall in cities, leaving the most car-dependent and low-household-income counties largely with lower coverage overall. The same trend continues when looking at fast charger coverage, with only two counties below the average household income threshold having fast charger coverage above 50%. When Alternative Fuel Corridors reach NEVI-compliant status, the majority of counties (94%) reach coverage above 75%. The counties left with coverage below 10% are very rural and represent a range of county-level median household incomes but are primarily car-dependent.

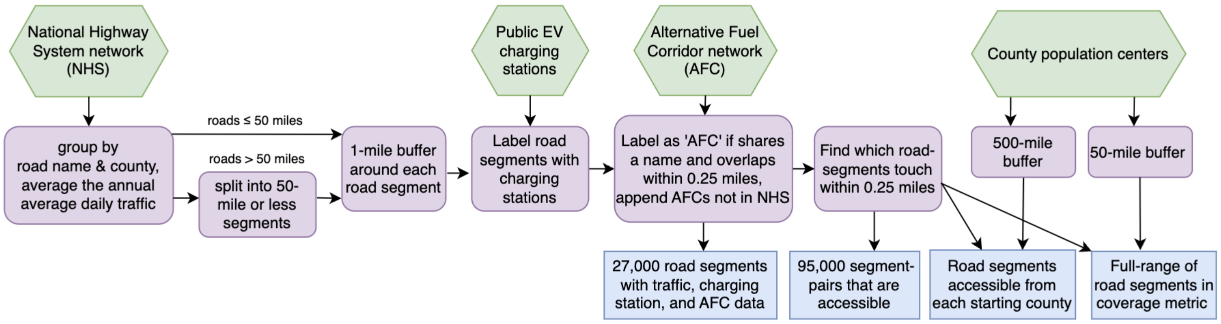

Supplementary Figure 7. **Data processing flow chart.** Flow of data processing before the breadth-first search function. Green hexagons represent data sources, purple bubbles represent a data processing step, and blue boxes represent an output used in the model.

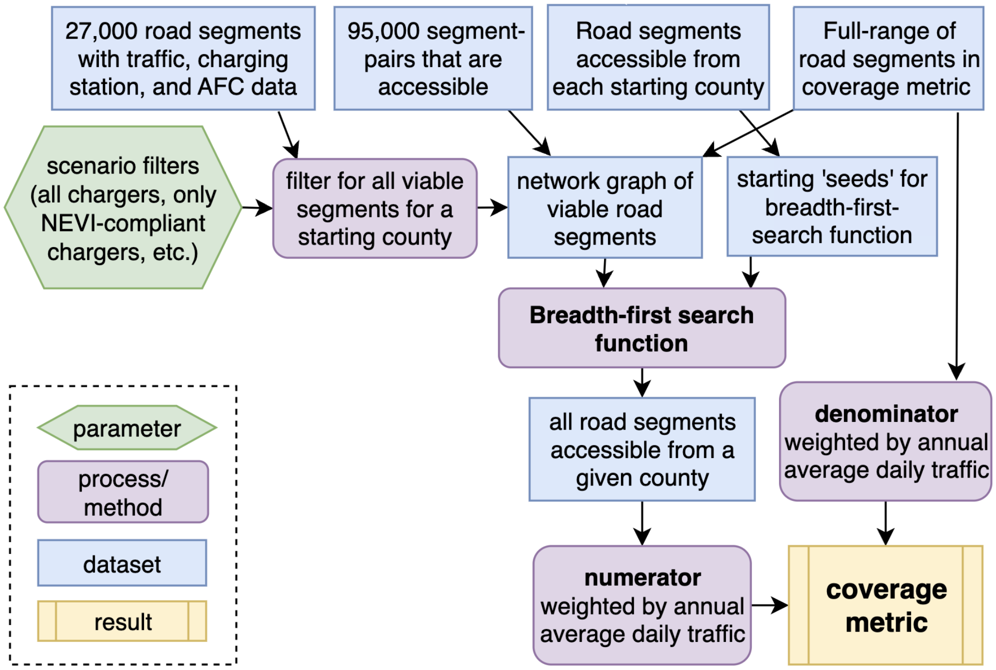

Supplementary Figure 8. **Flowchart of Methods.** for the coverage metric calculation with datasets used in blue, input parameters and assumptions in green, analysis methods in purple, and the final result outputs in blue. The National Highway System (NHS) dataset has 479,000 chunks of roads in the United States. We group road chunks into segments of each road by road name and county it passes through and average the annual average daily traffic for each road section. We label each road segment as containing an EV charging station (existing or planned) if a charging station falls within a 1-mile buffer (1,609.34 meters) of the segment (the National Electric Vehicle Infrastructure program guidelines require charging stations to be located within 1 mile of a given road). The Alternative Fuel Corridor (AFC) dataset did not geospatially align precisely with the NHS dataset; therefore, NHS roads were labeled as 'AFC' if an AFC road fell within 0.25 miles (400 meters) of a given road segment and shared the same name. Some roads (31 roads across 11 states) are AFCs but are not in the NHS dataset; any AFC roads not in the AFC dataset were assigned the average traffic of its connecting road segments and appended to the NHS dataset. Road segments are considered touching (i.e., accessible to each other) if they fall within 0.25 miles (400 meters) of each other.

Supplementary Discussion

Charging stations can be temporarily unavailable due to the need for repairs[5]. Charging station data were pulled from the U.S. Department of Energy Alternative Fuels Data Center[6] on March 1, 2023, and stations that came onto the U.S. Department of Energy fuel station tracker following March 1, 2023, are not included, nor are any stations listed as offline or not operational. However, there is a risk that stations included in this analysis could be offline or out of service at future times. Rempel et al. (2023) found that in the San Francisco area, only 73% of charging station ports were operating at any given time[7]. We do not account for charging stations dynamically going temporarily offline and back online. Future work could extend our model to stochastically account for the risk of a station going out of service.

In the fast charger and full NEVI-compliance scenarios, we only consider charging stations with at least four DC fast chargers. However, even a charging station with four DC fast chargers could have queuing or long wait times with high EV adoption and high traffic. We do not account for queuing directly in the model beyond requiring four charging stations. Future work could be done to extend this model to include not just consecutive coverage, but also consecutive coverage with a sufficient number of chargers per charging station given the annual average daily traffic and anticipated EV adoption. Only 1% of vehicles are EVs in the United States[8]; therefore, in the short term, queuing at stations on long-distance trips may pose an inconvenience, but not prevent the trip from being viable (the vehicle would have to wait to charge until a charger is free, but will be able to charge). The NEVI requirements of four DC fast chargers that can typically charge an EV in under an hour should be sufficient for near-term outlooks of EVs on the road.

Future research or extensions of this work could calculate the consecutive coverage metric, weighting road segments by peak traffic per year or congestion levels. Another extension of this paper could dynamically define the minimum number of chargers per station needed by the annual average daily traffic or peak daily traffic of the year (i.e., requiring more charging ports for high-trafficked roads), therefore identifying not only gaps in coverage, but segments of road that contain an insufficient number of chargers per station. As more consumers adopt EVs, queuing times and high-congestion travel days may become a primary form of charging anxiety. Therefore, extending this work to look specifically at peak congestion both for weighting the road segments in the coverage metric and considering the sufficiency of chargers per station would be a valuable extension of this work.

Future work could look at the trade-offs in EV adoption impacts between investing in community-level charging station access vs. long-distance charging station coverage. An optimization of community-level and long-distance charging needs could yield a Pareto frontier of ideal charging station distribution between local access and long-distance coverage. Additionally, models of EV adoption, such as NREL's TEMPO model, could be used to extend consecutive coverage work to understand the influence on EV adoption by charging station coverage and the resulting change in lifecycle emissions from higher EV adoption.

Supplementary References

1. Insider, B. *What EV owners need to know about Tesla's charging partnership with Ford and GM* https://www.businessinsider.com/how-teslas-charging-partnership-ford-and-gm-works-consumers-2023-6.
2. North American Council for Freight Efficiency. *The Case for MD Box Trucks* https://nacfe.org/research/run-on-less/run-on-less-electric/md-box-trucks/.
3. Borlaug, B. *et al.* Charging needs for electric semi-trailer trucks. *Renewable and Sustainable Energy Transition* **2,** 100038. ISSN: 2667-095X. doi:https://doi.org/10.1016/j.rset.2022.100038. https://www.sciencedirect.com/science/article/pii/S2667095X22000228 (2022).
4. U.S. Department of Energy. *National Alternative Fuels Corridors* https://afdc.energy.gov/laws/11675.
5. Hawkins, A. J. *Electric vehicle owners are fed up with broken EV chargers and janky software* https://research-hub.nrel.gov/en/publications/quantifying-the-tangible-value-of-public-electric-vehicle-chargin.
6. U.S. Department of Energy. *Alternative Fueling Station Locator* https://afdc.energy.gov/stations#/find/nearest.
7. Rempel, D., Cullen, C., Bryan, M. & Cezar, G. V. Reliability of Open Public Electric Vehicle Direct Current Fast Chargers. *Human Factors* **66,** 00187208231215242. doi:10.1177/00187208231215242 (2023).
8. US Department of Energy. *Electric Vehicles Registered in 2021* https://afdc.energy.gov/transatlas/#/?view=percent&state=US.
